# Medium-sized follicle proportion on the trigger day may be associated with higher live birth rate in fresh embryo transfer cycles among low-prognosis patients: a retrospective cohort study

**DOI:** 10.3389/fendo.2026.1888870

**Published:** 2026-07-15

**Authors:** Huiqun Yin, Jie Zhu, Cunli Wang, Kang Luan, Yan Wu, Feng Ni

**Affiliations:** 1 Reproductive Medicine Center, The 901st Hospital of PLA Joint Logistics Support Force, Hefei, China; 2 Reproductive Medicine Center, Anhui No.2 Provincial People’s Hospital, Hefei, China; 3 Anhui Institute of Medicine, Hefei, China

**Keywords:** follicle size, GnRH antagonist protocol, live birth rate, low prognosis, POSEIDON criteria

## Abstract

**Objective:**

To investigate the association of medium-size follicle proportions (MFP) on the trigger day with the pregnancy outcomes in low prognosis patients undergoing GnRH antagonist protocol.

**Materials and methods:**

A total of 1084 oocyte-retrieval cycles from 765 patients diagnosed with low prognosis according to POSEIDON criteria were enrolled in this retrospective study between January 2017 and December 2023. MFP was defined as ratio of the number of follicles sized 13–18 mm to the number of follicles ≥ 12 mm on trigger day. According to the value of MFP, cycles were divided into MFP < 70% and MFP ≥ 70% groups. Cycle characteristics, laboratory data and pregnancy outcomes were compared between the two groups. Multivariate logistic regression analysis was used to identify whether the value of MFP was independent predictor of live birth.

**Results:**

The live birth rate (LBR) (20.75% vs 31.88%, P = 0.003) in fresh embryo transfer cycles was significantly lower in the MFP < 70% group than in the MFP ≥ 70% group. This difference was observed both in patients aged ≤ 35 years (28.18% vs 43.28%, P = 0.0148) and > 35 years (14.50% vs 23.66%, P = 0.0444). A significant increase in GnRH-antagonist dose (0.91 ± 0.44 mg vs 0.78 ± 0.43 mg, P < 0.0001) and duration (3.61 ± 1.57 days vs 3.11 ± 1.55 days, P < 0.0001) was observed in MFP < 70% group. No significant difference was observed in the LBR between groups in frozen-thawed embryo transfer (FET) cycles. Multivariate logistic regression analysis showed that the value of MFP was positively associated with live births in fresh transfers whether MFP was modeled as a continuous variable (per 1% increase) or as a binary threshold (≥ 70% vs. < 70%).

**Conclusions:**

These findings highlight the significant association of MFP with LBR in fresh transfer cycles, suggesting that more attention should be paid to medium-sized follicle development when determining the timing of trigger and embryo transfer strategies for low prognosis patients. No significant association was observed in FET cycles, suggesting that the benefit of higher MFP may be specific to fresh transfer.

## Introduction

Gonadotropin releasing hormone (GnRH) antagonists are widely used for ovarian stimulation due to their safety and flexibility, including in poor prognosis patients (1). Determining the optimal time for ovulation triggering remains challenging, as follicle size is a key predictor of metaphase II (MII) oocyte yield (2). The decision involves considering the size distribution of the growing follicle cohort, hormonal levels, duration of stimulation, patient burden, and experience of previous cycles (3). Although criteria often rely on the presence of large follicles (≥ 17–18 mm), follicles outside an optimal size range are less likely to yield oocytes (4, 5), and the relationship between follicle size and pregnancy outcomes remains inadequately studied (3, 6–8). Notably, a multi-center study based on machine learning analysis has shown that follicles measuring 13–18 mm contribute most to MII oocyte yield and live birth rate (LBR), with benefits plateauing when ≥ 70% of follicles fall within this range. Larger follicles (> 18 mm) have been associated with premature progesterone elevation and reduced fresh cycle LBR (2). While dominant follicles (> 18 mm) are linked to premature progesterone elevation, synchronization within the 13–18 mm range may optimize oocyte maturity while reducing endometrial disruption. We thus hypothesize that MFP ≥ 70% reflects follicular homogeneity, potentially enhancing fresh-transfer outcomes via endometrial receptivity pathways.

POSEIDON (patient-oriented strategies encompassing individualized oocyte number) criteria define low-prognosis patients as those with poor ovarian response (≤ 9 oocytes retrieved) or diminished ovarian reserve (antral follicle count (AFC) < 5 or anti-Müllerian hormone (AMH) level < 1.2 ng/mL) (9), representing nearly 40% of IVF patients (10). These individuals exhibit distinct follicular growth patterns (11) and are challenging to manage. Recent studies suggest that a high proportion of dominant follicles may reduce oocyte yield and available embryos (4), and frozen embryo transfer (FET) may lead to lower LBR compared to fresh transfers in this group (12). Therefore, maximizing the quality and quantity of embryos derived from the limited oocytes obtained, as well as optimizing embryo transfer strategies, are crucial for these patients, as they directly impact patient-centered outcomes (13).

In GnRH antagonist protocols, the antagonist is initiated after gonadotropin (Gn) stimulation has already begun, and its primary role is to prevent premature luteinizing hormone (LH) surges. Consequently, follicular recruitment is largely determined by the Gn stimulation itself, while the timing of antagonist initiation and trigger day primarily influences the final maturation and synchrony of the developing follicle cohort. For low-prognosis patients, who have a limited pool of recruitable follicles, optimizing the size distribution at trigger is particularly critical, as it directly affects the likelihood of retrieving mature oocytes and, ultimately, the number of available embryos. Therefore, understanding the relationship between follicular size distribution on the trigger day and oocyte competence is of substantial clinical relevance in this population.

Based on this, we hypothesized that a medium-sized follicle proportion (MFP) ≥ 70% on the trigger day is associated with improved live birth outcomes in fresh embryo transfer cycles among low prognosis patients undergoing GnRH antagonist protocols. And the value of MFP may be an influencing factor on live birth for fresh embryo transfer. Therefore, this retrospective study aims to evaluate the association of MFP on pregnancy outcomes in both fresh and first frozen embryo transfer cycles, to guide clinical trigger timing and embryo transfer strategies.

## Materials and methods

### Study subjects

This retrospective cohort study included oocyte retrieval cycles performed between January 2017 and December 2023. Eligible patients were aged 20–42 years, diagnosed with low prognosis undergoing GnRH antagonist protocol. Low prognosis was defined according to the POSEIDON criteria (groups 1–4) using data from the first oocyte-retrieval cycle of each patient. Patients with AFC <5, AMH <1.2 ng/mL, or ≤9 oocytes retrieved in the first cycle were considered to have diminished reserve or poor response. Exclusion criteria were: ① polycystic ovarian syndrome, hydrosalpinx, uterine malformation, history of intrauterine adhesions or recurrent clinical pregnancy loss; ② chromosome abnormality; ③ cancellation of follicular puncture; ④ preimplantation genetic testing cycle. This study adhered to the principles of the Declaration of Helsinki and was approved by the Ethics Committee, which waived written informed consent due to retrospective study (No.202504009).

MFP was defined as the ratio of the number of follicles sized 13–18 mm to the number of follicles ≥ 12 mm in diameter visualized on ultrasound on the trigger day. Based on recent findings utilizing artificial intelligence (2), we stratified cycles using an MFP threshold of 70%, resulting in MFP ≥ 70% and MFP < 70% groups. The 70% threshold was derived from the inflection point of the dose-response curve in the study by Hanassab et al. (2), showing plateaued benefit beyond this value. Cycle characteristics, serum hormone levels, oocyte and embryo development parameters, pregnancy outcomes of fresh embryo transfer and the first subsequent FET cycles were compared between the two groups.

### Ovarian stimulation protocol

On menstrual cycle day 2 or 3, ovarian reserve assessments (AFC, serum levels of AMH and basal follicle-stimulating hormone (FSH), LH, estradiol (E2), and progesterone) were performed prior to ovarian stimulation initiation. All patients underwent a flexible GnRH antagonist protocol. The initial Gn dose (150–300 IU) was individualized based on age, body mass index (BMI), AFC and AMH levels. Daily Gn injections (recombinant FSH (r-FSH; GONAL-f, Merck Serono, Switzerland; PUREGON, Merck Sharp & Dohme, Germany) and/or human menopausal gonadotropin (HMG; Menotropins for Injection, Lizhu Pharm, China)) commenced on cycle day 2 or 3. The Gn dose was adjusted based on follicular development as monitored by ultrasound and serum hormone levels. When the leading follicle reached 12–14 mm in diameter, or serum LH level showed a noticeable upward trend, daily subcutaneous administration of 0.25 mg GnRH antagonist (Cetrorelix acetate; Merck Serono, Switzerland) was initiated and continued until the trigger day.

Trigger timing was determined based on the diameter and number of dominant follicles and hormone levels. Final oocyte maturation was triggered with either using 250 μg of recombinant human chorionic gonadotropin (r-HCG; Serono) alone, or a dual trigger comprising 0.2 mg GnRH agonist (triptorelin 0.1 mg; Ferring Pharmaceuticals) and 2000 IU urinary HCG (u-HCG; Lizhu Pharm, China), when at least one follicle reached ≥ 18 mm or two follicles ≥ 17 mm in diameter.

### Oocyte retrieval and embryo culture

Oocytes were retrieved approximately 36 hours after HCG administration under transvaginal ultrasound guidance. Follicles approximately 12 mm or greater in diameter were aspirated. Oocytes were fertilized via conventional IVF or intracytoplasmic sperm injection (ICSI) based on semen analysis results. Fertilized oocytes were individually cultured in G1-plus media (Vitrolife, Gothenburg, Sweden) under paraffin oil at 37°C, 6% CO_2_ and 5% O_2_. Fertilization was assessed by the presence of pronuclei 18–20 hours post-insemination. Embryo quality was assessed using previously described standards (14). A good-quality embryos was defined as normal fertilized embryos with 2–5 cells on day 2 (D2), or > 6 cells on day 3 (D3), exhibiting < 25% fragmentation. Available embryos were defined as normal fertilized embryos with 2–6 cells on day 2 (D2), or ≥ 6 cells on day 3 (D3), exhibiting < 50% fragmentation. Embryos with ≥ 4 cells and < 50% fragmentation were eligible for extended culture to the blastocyst stage. A good-quality blastocyst was defined as having a well-expanded blastocoel (3–6) on day 5 or (4–6) on days 6, a well-defined inner ICM (A or B) and a single layer of trophectoderm cells surrounding the cavity (A or B). Available blastocysts were superior to grade 3CC (expansion grade of 3-6, ICM and TE grade of AA, AB, BA, BB, BC, CB) on day 5 or on day 6 according to Gardner criteria. Embryo cryopreservation was performed using vitrification method.

### Embryo transfer and luteal phase support

Fresh embryo transfer was performed only if all the following criteria were met on the trigger day: serum progesterone <1.5 ng/mL, absence of intrauterine fluid or polyps, and no clinical signs of ovarian hyperstimulation syndrome. Based on the number of available embryos, patient characteristics and preference, 1–3 available cleavage-stage embryos (D2 or D3), or 1–2 available blastocysts (D5) were transferred.

FET was performed for patients undergoing a ‘freeze-all’ strategy or those who did not achieve a live birth after fresh transfer. Endometrial preparation utilized natural cycles or hormone replacement cycles, tailored to patient characteristics and preferences. Each patient received 1–3 thawed D3 embryos, or 1–2 thawed blastocysts, or sequential transfer of one D3 embryo and one blastocyst.

Luteal phase support (LPS) consisted of daily intramuscular administration progesterone (40 mg; Progesterone Injection, Jinyao Tianjin, China) and oral dydrogesterone (20 mg; Dydrogesterone Tablets, Abbott Biologicals B.V., Netherlands). If serum β-HCG was positive two weeks after embryo transfer, LPS was continued to 10^th^ week of gestation.

### Pregnancy outcome parameters

The primary outcome was live birth, defined as the delivery of a live infant at ≥ 28 weeks of gestation. Secondary outcomes included clinical pregnancy, early miscarriage, ectopic pregnancy and implantation rate (IR). Clinical pregnancy was confirmed by the observation of a gestational sac under ultrasound on 30–35 days after embryo transfer. Early miscarriage was defined as pregnancy loss before 12 completed gestational weeks. IR was calculated as the number of gestational sacs observed divided by the number of transferred embryos.

### Statistical analysis

Continuous variables are presented as mean ± standard deviation (SD), and categorical variables are presented as a percentage. Comparisons between groups were performed using Pearson’s chi-square test, Fisher’s exact test, or Student’s t-test, as appropriate. Multivariate logistic regression analysis was used to identify independent predictors of live birth, adjusting for potential confounders. Variables were selected based on a combination of clinical relevance and the results of univariable analyses including female age, AMH, AFC, BMI, endometrium thickness, MFP value, the dose and duration of antagonist administration, the number of oocytes retrieved and transferred embryos, the number of previous oocyte-retrieval cycles. MFP value was entered as a continuous variable (per 1% increase) in the primary regression model to preserve its full granularity. To complement the continuous approach and to maintain clinical interpretability, a sensitivity analysis was performed in which MFP was entered as a binary variable (≥ 70% vs. < 70%) using the same covariate adjustment. The discriminatory ability of the final model was evaluated by the area under the receiver operating characteristic curve (AUC), and calibration was assessed using the Hosmer–Lemeshow goodness-of-fit test. To account for within−patient correlation and potential changes in ovarian reserve over time, a complete sensitivity analysis was performed restricted to the first oocyte−retrieval cycle. And the same models were re-run only for D3-embryo transfers to confirm the robustness of the associations. Statistical analyses were conducted using Prism 9.0. Power analyses for pregnancy outcomes of FET cycles were calculated using OpenEpi Version 3.01 (http://www.openepi.com/Menu/OE_Menu.htm). Two-sided 95% confidence intervals (CI) were reported. A P values < 0.05 were considered statistically significant.

## Results

### Baseline characteristics and overall pregnancy outcomes of patients between the MFP ≥ 70% and MFP < 70% groups

A total of 1084 oocyte-retrieval cycles from 765 low-prognosis patients undergoing the GnRH antagonist protocol were included. Baseline characteristics of patients assessed at the first oocyte-retrieval cycle are presented in **Table 1**. No significant differences were observed between the MFP < 70% and MFP ≥ 70% groups in age, infertility duration, base FSH, AMH, AFC and primary infertility ratio. Among the 765 patients, the distribution according to the POSEIDON criteria based on the first oocyte-retrieval cycle was as follows: Group 1, 16.60% (n=127); Group 2, 20.79% (n=159); Group 3, 22.48% (n=172); Group 4, 40.13% (n=307) (**Supplementary Table 1**). The proportion of patients with MFP ≥ 70% did not differ significantly across the four POSEIDON groups (P = 0.1940).

**Table 1:** Baseline characteristics of patients at the first oocyte-retrieval cycle stratified by the medium-sized follicles proportion (MFP).

	MFP < 70%	MFP ≥ 70%	P value
Patients (n)	319	446	/
Female age (years)	34.93 ± 4.78	35.46 ± 4.84	0.1014
Infertility duration (years)	4.32 ± 3.58	4.84 ± 4.01	0.0982
Primary infertility ratio (%)	27.90 (89/319)	32.06 (143/446)	0.2168
BMI (kg/m^2^)	23.39 ± 3.24	23.58 ± 3.67	0.7932
Base FSH (IU/L)	10.72 ± 5.34	10.46 ± 5.46	0.5031
AMH (ng/mL)	0.95 ± 0.52	0.96 ± 0.59	0.8281
AFC (n)	5.0 ± 2.65	5.14 ± 3.46	0.4437

AFC, antral follicle count; AMH, anti-Müllerian hormone; BMI, body mass index; FSH, follicle stimulating hormone.

Based on MFP on the trigger day, 448 cycles (41.33%) were assigned to the MFP < 70% group and 636 cycles (58.67%) to the MFP ≥ 70% group. A total of 326 and 459 embryo transfer cycles including fresh transfers and first FET occurred in the MFP < 70% and ≥ 70% groups, respectively. Overall pregnancy outcomes showed a significantly lower LBR (22.39% vs 29.85%, P = 0.0201) and a higher early miscarriage rate (EMR) (28.57% vs 16.76%, P = 0.0167) in the MFP < 70% group compared to the MFP ≥ 70% group. No significant difference was observed in the overall clinical pregnancy rate (CPR).

### Ovarian stimulation and laboratory data between the MFP ≥ 70% and MFP < 70% groups

Cycle characteristics and laboratory data of all fresh oocyte-retrieval cycles are compared in **Table 2**. Compared to the MFP ≥ 70% group, the MFP < 70% group exhibited a significantly higher dose (0.91 ± 0.44 mg vs 0.78 ± 0.43 mg, P < 0.0001) and longer duration (3.61 ± 1.57 days vs 3.11 ± 1.55 days, P < 0.0001) of GnRH antagonist administration, and a lower mean oocyte retrieval rate per follicle ≥ 12 mm (1.02 ± 0.37 vs 1.09 ± 0.46, P = 0.0422). Because oocytes retrieved from follicles < 12 mm were included in the numerator but the corresponding follicles were not counted in the denominator, the oocyte retrieval rate could be greater than 1. Regarding oocyte and embryo development, laboratory outcomes were generally comparable between groups, including the number of oocytes retrieved, number of available embryos, MII oocyte rate in ICSI cycles, and the rate of cycles with no available embryo (P > 0.05). The exception was a lower MII oocyte rate in IVF cycle in the MFP < 70% group (79.07% vs 82.89%, P = 0.0054).

**Table 2:** Controlled ovarian hyperstimulation cycles and laboratory outcomes stratified by the medium-sized follicles proportion (MFP).

	MFP < 70%	MFP ≥ 70%	P value
No. of oocyte-retrieval cycles (n)	448	636	/
Total Gn dose (IU)	2305 ± 923.7	2264 ± 878.1	0.6658
Duration of Gn (days)	8.56 ± 2.44	8.40 ± 2.21	0.4184
Total GnRH-ant dose (mg)	**0.91 ± 0.44**	**0.78 ± 0.43**	**< 0.0001**
Duration of GnRH-ant (days)	**3.61 ± 1.57**	**3.11 ± 1.55**	**< 0.0001**
E2 level/follicle (≥12 mm) on the trigger day (pg/mL)	264.3 ± 124.7	272.1 ± 131.6	0.4398
Progesterone on the trigger day (ng/mL)	0.84 ± 0.48	0.84 ± 0.61	0.3285
LH on the trigger day (IU/L)	4.32 ± 3.47	4.43 ± 4.37	0.1148
Trigger drugs (%)a	/	/	0.9498
HCG	87.50 (392/448)	87.11 (554/636)	/
GnRHa	0.22 (1/448)	0.31 (2/636)	/
HCG + GnRHa	12.28 (55/448)	12.58 (80/636)	/
Mean oocyte retrieval rate	**1.02 ± 0.37**	**1.09 ± 0.46**	**0.0422**
Fertilization method (%)b	/	/	0.7646
IVF	71.65 (321/448)	72.17 (459/636)	/
ICSI	26.56 (119/448)	25.47 (162/636)	/
IVF + rescue-ICSI	1.79 (8/448)	2.36 (15/636)	/
No. of oocyte retrieved (n)	4.42 ± 2.17	4.28 ± 2.33	0.2195
Ratio of MII oocyte in ICSI cycles (%)	76.50(394/515)	75.03(574/773)	0.5466
Ratio of MII oocyte in IVF cycles (%)	**79.07 (1122/1419)**	**82.89 (1555/1876)**	**0.0054**
Fertilization rate in ICSI cycle (%)	85.03(335/394)	84.67(490/574)	0.8794
Fertilization rate in IVF cycle (%)	67.79(962/1419)	70.90(1345/1876)	0.0554
No. of available embryo (n)	2.14 ± 1.09	2.03 ± 1.12	0.0868
Ratio of available embryo in IVF cycle (%)	58.94 (590/1001)	59.15 (756/1278)	0.9179
Ratio of no available embryo cycle (%)	15.33 (69/448)	17.14 (109/636)	0.4286

AFC, antral follicle count; AMH, anti-Müllerian hormone; BMI, body mass index; E2, oestradiol; FSH, follicle stimulating hormone; Gn, gonadotropin; GnRH-ant, gonadotropin releasing hormone antagonist; HCG, human chorionic gonadotropin; ICSI, intracytoplasmic sperm injection; IVF, *in-vitro* fertilization. LH, luteinizing hormone; MFP, medium-size follicle proportions; MII, metaphase II. a Chi-square test showed no significant between‑group difference in trigger drug proportions. GnRHa-only trigger (0.2 mg triptorelin acetate) was used in only 3 cycles. b Chi-square test showed no significant between‑group difference in fertilization method proportions.

### Pregnancy outcomes of fresh embryo transfer cycles between the MFP < 70% and MFP ≥ 70% groups

Among all oocyte-retrieval cycles, fresh transfer was performed in 53.79% (241/448) of the MFP < 70% group and 50.31% (320/636) of the MFP ≥ 70% group (P = 0.2588). The most common reasons for not proceeding to fresh transfer were no transferrable embryos (39.13% vs. 35.76%) and “freeze-all” strategy including progesterone elevation exceeding 1.5 ng/mL (12.08% vs. 8.86%), uterine cavity and endometrial factors (23.19% vs. 28.80%), planned double stimulation (10.63% vs. 11.71%), patient−requested “freeze−all” (10.14% vs. 8.86%) and other factors (infection, diarrhea, incomplete documentation, etc., 4.83% vs. 6.01%); none of which differed significantly between the two groups (all P > 0.05).

**Figure 1:**
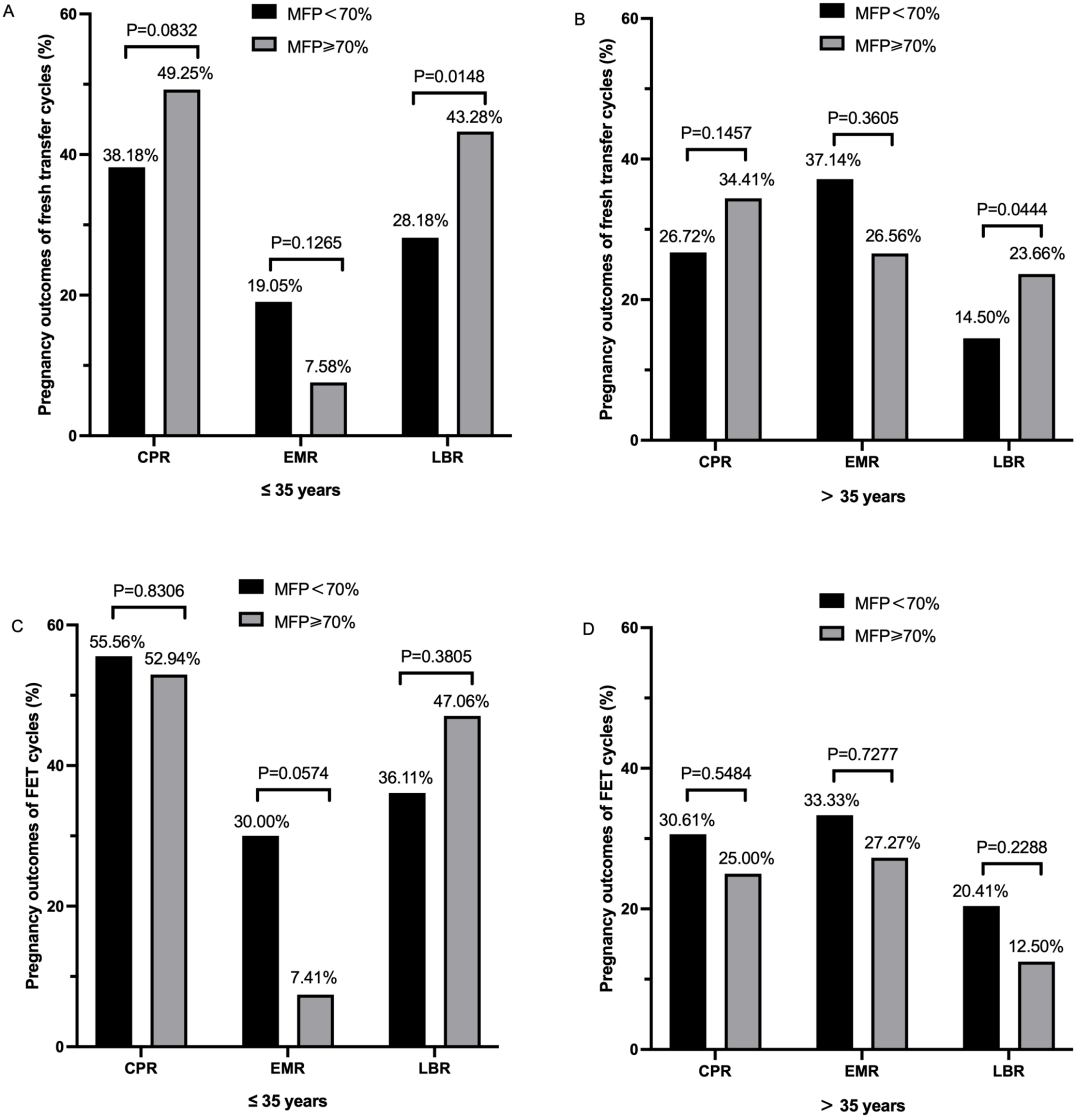
Pregnancy outcomes were compared between the MFP < 70% and MFP ≥ 70% groups in fresh embryo transfer cycles **(A, B)** and the first FET cycles **(C, D)**, stratified by age (≤ 35 years and > 35 years). **(A)** Clinical pregnancy rate (CPR) and live birth rate (LBR) in fresh transfer cycles (≤ 35 years). **(B)** CPR and LBR in fresh transfer cycles (> 35 years). **(C)** CPR and LBR in FET cycles (≤ 35 years). **(D)** CPR and LBR in FET cycles (> 35 years). Abbreviations: MFP, medium-sized follicle proportion; FET, frozen-thawed embryo transfer; CPR, clinical pregnancy rate; LBR, live birth rate; EMR, early miscarriage rate.

Clinical outcomes of the 241 fresh embryo transfer cycles in the MFP < 70% group and 320 cycles in the MFP ≥ 70% group are shown in **Table 3**. Both CPR (31.95% vs 40.63%, P = 0.035) and LBR (20.75% vs 31.88%, P = 0.003) were significantly lower in the MFP < 70% group compared to the MFP ≥ 70% group. IR and EMR did not differ significantly. Due to the limited sample sizes in D2 (n = 47 vs. 40) and D5 (n = 20 vs. 21) subgroups, the comparisons were underpowered; no firm conclusions can be drawn. However, for D3 embryo transfers, both CPR (34.48% vs 44.02%, P = 0.0473) and LBR (21.84% vs 33.98%, P = 0.0064) were significantly lower in the MFP < 70% group. Further stratification by age showed that the lower LBR in the MFP < 70% group was consistent in both patients ≤ 35 years (28.18% vs 43.28%, P = 0.0148) and > 35 years (14.50% vs 23.66%, P = 0.0444). CPR and EMR did not differ significantly by age group (**Figures 1A, B**).

**Table 3:** Clinical outcomes of fresh embryo transfer stratified by the medium-sized follicles proportion (MFP).

	MFP < 70%	MFP ≥ 70%	P value
Fresh transfer cycles (n)	241	320	/
Female age (years)	35.50 ± 5.03	35.67 ± 4.90	0.7865
Total Gn dose (IU)	2356 ± 942.4	2302 ± 812.1	0.7420
Duration of Gn (days)	8.66 ± 2.27	8.58 ± 2.05	0.5437
Total GnRH-ant dose (mg)	**0.95 ± 0.41**	**0.82 ± 0.41**	< 0.0001
Duration of GnRH-ant (days)	**3.78 ± 1.55**	**3.27 ± 1.48**	< 0.0001
Progesterone on the trigger day (ng/mL)	0.75 ± 0.36	0.72 ± 0.33	0.3481
Endometrium thickness (mm)	10.19 ± 2.27	9.92 ± 2.41	0.1267
No. of oocyte retrieved (n)	4.92 ± 2.08	4.82 ± 2.13	0.5808
No. of embryos transferred (n)	**1.85 ± 0.64**	**1.68 ± 0.59**	**0.0029**
D2-embryo transfer	**2.38 ± 0.53**	**2.08 ± 0.42**	0.0038
D3-embryo transfer	1.76 ± 0.60	1.67 ± 0.58	0.1012
Blastocyst transfer	1.30 ± 0.47	1.10 ± 0.30	0.1300
Transferred embryo stage (%) a	/	/	0.0434
D2-embryo transfer	19.50 (47/241)	12.50 (40/320)	**0.0233**
D3-embryo transfer	72.20 (174/241)	80.94 (259/320)	**0.0146**
Blastocyst transfer	8.30 (20/241)	6.56 (21/320)	0.5127
Cycle with at least one good-quality embryo (%) b	95.02 (229/241)	96.56 (309/320)	0.3620
D2-embryo transfer	95.74 (45/47)	97.50 (39/40)	> 0.9999
D3-embryo transfer	95.98 (167/174)	96.91 (251/259)	0.6032
Blastocyst transfer	85.00 (17/20)	90.48 (19/21)	0.6628
CPR	**31.95 (77/241)**	**40.63 (130/320)**	**0.0350**
D2-embryo transfer	25.53 (12/47)	22.50 (9/40)	0.8052
D3-embryo transfer	**34.48 (60/174)**	**44.02 (114/259)**	**0.0473**
Blastocyst transfer	25.00 (5/20)	33.33 (7/21)	0.7337
IR	21.57 (96/445)	26.95 (145/538)	0.0510
D2-embryo transfer	12.50 (14/112)	10.84 (9/83)	0.7229
D3-embryo transfer	25.08 (77/307)	29.86 (129/432)	0.1533
Blastocyst transfer	19.23 (5/26)	30.43 (7/23)	0.5082
EMR	27.27 (21/77)	16.92 (22/130)	0.0761
D2-embryo transfer	16.67 (2/12)	11.11 (1/9)	> 0.9999
D3-embryo transfer	30.00 (18/60)	17.54 (20/114)	0.0587
Blastocyst transfer	20.00 (1/5)	14.29 (1/7)	> 0.9999
LBR	**20.75 (50/241)**	**31.88 (102/320)**	**0.0033**
D2-embryo transfer	17.02 (8/47)	20.00 (8/40)	0.7855
D3-embryo transfer	**21.84 (38/174)**	**33.98 (88/259)**	**0.0064**
Blastocyst transfer	20.00 (4/20)	28.57 (6/21)	0.7186
Ectopic pregnancy	2	1	/

Group sample sizes for age stratification: ≤ 35y (MFP < 70%: n = 109; MFP ≥ 70%: n = 134). CPR, clinical pregnancy rate; D2, day 2; D3, day 3; EMR, early miscarriage rate; Gn, gonadotropin; GnRH-ant, gonadotropin releasing hormone antagonist; IR, implantation rate; LBR, live birth rate; MFP, medium-size follicle proportions. a Chi‑square test showed significant between‑group difference in the proportion of cycles by transferred embryo stage (p < 0.05). b Chi-square test showed no significant between-group difference in the proportion of all cycles including D2, D3 and blastocyst transfer with at least one good-quality embryo.

Multivariate logistic regression analysis (**Table 4**) identified female age as negatively associated, AFC, MFP value (adjusted OR = 1.02, 95% CI 1.01-1.03, P = 0.0020), endometrial thickness as positively associated with live births in fresh embryo transfer cycles. The number of previous retrieval cycles was not associated with live births. After adding the number of transferred embryos to the regression model for live birth, this variable became significant (adjusted OR = 1.94, 95% CI 1.30-2.94, P = 0.0015), whereas the number of oocytes retrieved lost significance (adjusted OR = 1.05, 95% CI 0.93-1.17, P = 0.4400). All variance inflation factor (VIF) values were below 2.5, indicating no significant multicollinearity. The final model for live birth demonstrated acceptable discrimination (AUC = 0.72, 95% CI 0.68–0.77, P < 0.0001) and good calibration (Hosmer–Lemeshow test P = 0.4732). In a sensitivity analysis of MFP as the binary threshold (MFP ≥ 70% vs. < 70%), the association remained significant (adjusted OR = 2.12, 95% CI 1.38-3.32, P = 0.0008). In a sensitivity analysis restricted to D3-embryo transfers whether MFP as a continuous variable (per 1% increase) or the binary threshold (MFP ≥ 70% vs. < 70%) in the primary model, the association remained significant (adjusted OR = 1.02, 95% CI 1.01-1.03, P = 0.0012; or adjusted OR = 2.26, 95% CI 1.39-3.75, P = 0.0012).

**Table 4:** Multivariate logistic regression analysis of factors associated with live births in all fresh embryo transfer cycles.

	OR (95% CI)	P value	Adjusted OR (95% CI)	P value
Female age (years)	**0.91 (0.88-0.95)**	**< 0.0001**	**0.90 (0.87-0.94)**	**< 0.0001**
AFC (n)	**1.14 (1.07-1.22)**	**< 0.0001**	**1.12 (1.04-1.20)**	**0.0016**
Endometrium thickness (mm)	**1.14 (1.06-1.24)**	**0.0009**	**1.11 (1.01-1.21)**	**0.0245**
MFP value (per 1% increase)	**1.01 (1.00-1.02)**	**0.0403**	**1.02 (1.01-1.03)**	**0.0020**
No. of retrieved oocytes (n)	**1.16 (1.06-1.27)**	**0.0013**	1.05 (0.93-1.17)	0.4400
No. of transferred embryos (n)	1.29 (0.95-1.75)	0.1007	**1.94 (1.30-2.94)**	**0.0015**
No. of previous retrieval cycles (n)	0.88 (0.74-1.02)	0.1140	1.01 (0.83-1.21)	0.8993
Transferred embryo stage	/	/	/	/
D3-embryo	Ref.	Ref.	Ref.	Ref.
D2-embryo	**0.55 (0.30-0.96)**	**0.0430**	0.48 (0.24-0.93)	**0.0341**
Blastocyst	0.79 (0.36-1.60)	0.5249	0.91 (0.37-2.10)	0.8369

MFP was entered as a continuous variable (per 1% increase) in the primary model. In a sensitivity analysis using the binary threshold (MFP ≥ 70% vs. < 70%), the association remained significant (adjusted OR = 2.12, 95% CI 1.38-3.32, P = 0.0008). All models were adjusted for the covariates listed. VIF values were < 2.5, indicating no significant multicollinearity. AFC, antral follicle count; CI, confidence intervals; MFP, medium-size follicle proportions; OR: odds ratio. Transferred embryo stage was categorized as D2, D3 (Ref., reference) and blastocyst. ORs were for each category relative to D3.

### Pregnancy outcomes of the first FET cycles between the MFP < 70% and MFP ≥ 70% groups

Clinical outcomes of the first FET cycles (85 cycles from MFP < 70% group, 139 cycles from MFP ≥ 70% group; excluding transfers from mixed retrieval cycles) are presented in **Table 5**. No significant differences were observed between groups in CPR, IR, or LBR overall. The *post-hoc* power calculations showed that the FET analysis had only 11.1% power to detect a difference in CPR, 5.6% for IR, and 1.5% for LBR with continuity correction. Stratification by age (≤ 35 vs > 35 years) also showed no significant differences in CPR, EMR, or LBR (**Figures 1C, D**).

**Table 5:** Clinical outcomes of the first frozen-thawed embryo transfer (FET) cycles stratified by the medium-sized follicles proportion (MFP).

	MFP < 70%	MFP ≥ 70%	P value
FET cycles (n)	85	139	/
Female age (years)	36.20 ± 4.09	36.40 ± 4.72	0.5136
Endometrium thickness (mm)	9.22 ± 2.27	9.23 ± 2.41	0.8977
No. of embryos transferred (n)	1.58 ± 0.61	1.50 ± 0.59	0.3391
Ratio of blastocyst transfer (%)	36.47 (31/85)	42.45 (59/139)	0.3761
CPR (%)	41.18 (35/85)	35.25 (49/139)	0.3741
D3-embryo transfer	34.62 (18/52)	21.92 (16/73)	0.1535
Blastocyst transfer	51.61 (16/31)	50.85 (30/59)	0.9450
Sequential transfer	50.00 (1/2)	42.86 (3/7)	> 0.9999
IR (%)	27.61 (37/134)	25.00 (52/208)	0.5910
Cleavage-embryo transfer	19.59 (19/97)	14.17 (18/127)	0.2796
Blastocyst transfer	50.00 (17/34)	46.97 (31/66)	0.7739
Sequential transfer	33.33 (1/3)	20.00 (3/15)	> 0.9999
EMR (%)	31.43 (11/35)	16.33 (8/49)	0.1194
LBR (%)	27.06 (23/85)	25.18 (35/139)	0.7554

Post−hoc power calculations showed low power for FET comparisons (CPR: 11.1%; IR: 5.6%; LBR: 1.5%); therefore, null findings should not be interpreted as evidence of no association. Group sample sizes for age stratification: ≤ 35y (MFP < 70%: n = 36; MFP ≥ 70%: n = 51). CPR, clinical pregnancy rate; EMR, early miscarriage rate; IR, implantation rate; LBR, live birth rate. CPR refers to the clinical pregnancy rate calculated from all cycles, including those with D3 embryos, blastocysts, and sequential embryo transfers. IR refer to the implantation rate calculated from all cycles, including those with D3 embryos, blastocysts, and sequential embryo transfers.

### Analysis of clinical outcomes and influencing factors restricted to the first oocyte-retrieval cycle per patient

To account for potential changes in ovarian reserve over time and to eliminate within patient correlation, we performed a supplementary analysis restricted to the first oocyte-retrieval cycle of each patient (n = 765 cycles). Characteristics of embryos transferred and clinical outcomes are summarized in **Supplementary Table 2**. The live birth rate was significantly lower in the MFP < 70% group compared with the MFP ≥ 70% group (21.35% vs. 37.50%, P = 0.0005), consistent with the main analysis.

In a multivariable logistic regression restricted to first-cycle fresh transfers (**Supplementary Table 3**), MFP as a continuous variable remained independently associated with live birth (adjusted OR = 1.02, 95% CI 1.01-1.03, P = 0.0013). When MFP was dichotomized at the 70% threshold, the association also remained significant (adjusted OR = 2.88, 95% CI 1.74-4.88, P < 0.0001). In a sensitivity analysis restricted to D3-embryo transfers using MFP as a continuous variable (per 1% increase) or using the binary threshold (MFP ≥ 70% vs. < 70%) in the primary model, the association remained significant (adjusted OR = 1.03, 95% CI 1.01-1.04, P = 0.0003; or adjusted OR = 3.21, 95% CI 1.82-5.83, P< 0.0001). These results confirm that the association between MFP and live birth is robust and not driven by repeated cycles.

## Discussions

This study demonstrates that an MFP ≥ 70% on the trigger day is significantly associated with higher LBR in fresh embryo transfers among low-prognosis patients undergoing GnRH antagonist protocols. This association persisted across age groups and was independent of female age and endometrial thickness. No such benefit was observed in frozen-thawed cycles.

This observed association between MFP and fresh-cycle live birth, contrasted with its absence in FET cycles, raises the hypothesis that MFP may influence the intra-cycle endometrial environment rather than embryo quality alone. First, follicular asynchrony may contribute to premature progesterone elevation during the late follicular phase, thereby impairing endometrial receptivity. Notably, although all fresh transfer patients in our cohort had progesterone <1.5 ng/mL on the trigger day, the MFP < 70% group still exhibited significantly lower live birth rates, suggesting that even subtle endocrine alterations associated with follicular asynchrony may adversely affect receptivity. This interpretation is supported by recent evidence demonstrating that in cycles with normal progesterone on the trigger day, transient progesterone elevation (≥1.5 ng/mL) occurring at any time prior to the trigger day is also associated with significantly lower pregnancy outcomes (15). Thus, patients with lower MFP may have experienced transient elevations during ovarian stimulation, prematurely advancing endometrial maturation despite normal levels on the trigger day. Second, GnRH antagonists may directly impair endometrial receptivity through multiple molecular mechanisms, including down-regulation of HOXA10, inhibition of c-kit-mediated stromal cell migration, induction of endometrial epithelial cell apoptosis, and epigenetic modifications such as Hoxa10 promoter methylation (16–21). These effects have been shown to be more pronounced with GnRH antagonist than with GnRH agonist protocols. However, we acknowledge that we did not measure direct biomarkers of endometrial receptivity (e.g., integrin β3, HOXA10, or endometrial transcriptomic profiles), and our multivariable analysis did not identify antagonist dose or duration as independent predictors. Therefore, this mechanistic interpretation remains speculative. We have framed it as a potential mechanism and a working hypothesis that requires validation in future studies specifically designed to assess endometrial receptivity markers in relation to follicular size synchrony.

Our finding of a lower mean oocyte retrieval rate per follicle ≥ 12 mm in the MFP < 70% group aligns with the concept that follicles within an optimal size range have the highest likelihood of yielding MII oocytes (2, 7). Triggering when a higher proportion of follicles fall within this range (MFP ≥ 70%) appears to optimize oocyte yield efficiency. The observed lower MII rate specifically in IVF cycles within the MFP < 70% group further supports the notion that suboptimal follicle size distribution may compromise oocyte maturity, particularly evident in conventional fertilization settings. However, it is noteworthy that once oocytes were retrieved and fertilized, the rates of fertilization and development into morphologically available embryos were similar between groups. This suggests that oocytes retrieved from cycles with lower MFP, although potentially less mature at retrieval (especially in IVF), can still achieve normal fertilization and early cleavage-stage development comparable to those from cycles with higher MFP.

Our results complement and extend previous findings. Xie et al. (4) also reported adverse outcomes associated with a high proportion of dominant (≥ 18mm) follicles in low-prognosis patients. Hanassab et al. (2) similarly found that larger mean follicle sizes, particularly > 18mm, were linked to premature progesterone elevation and reduced fresh transfer LBR. Our focus on the MFP provides a different perspective, emphasizing follicular homogeneity within the optimal size range rather than the dominance of large follicles. We observed that triggering when MFP is high (≥ 70%), implying a more synchronous cohort within the 13-18mm range, is beneficial for fresh LBR. This contrasts with a previous study that found delaying trigger to allow medium follicle growth did not improve outcomes (22), potentially highlighting the importance of the proportion at trigger rather than merely the presence of medium follicles. Recent randomized evidence from Wei et al. (12) suggested that FET may not universally confer benefit over fresh transfer in low-prognosis patients, depending on the number of oocytes retrieved and blastocyst availability. Our findings add to this discourse by demonstrating that fresh transfer outcomes are modifiable through optimizing follicular synchrony at trigger, which could inform individualized transfer strategies.

Subgroup analyses by specific transfer types (particularly Day 2 fresh transfers, blastocyst fresh transfers, and sequential FET transfers) suffered from limited sample sizes and correspondingly low statistical power. Therefore, our primary conclusions are based on the overall fresh transfer population and the Day 3 subgroup. Moreover, we analyzed MFP both as a continuous variable and as a dichotomized variable. The continuous analysis provides statistical precision, while the binary threshold offers practical guidance for clinicians at the bedside. The concordant results between the two methods reinforce our confidence in the association.

We observed that the MFP < 70% group had a significantly higher number of embryos transferred, especially in D2-embryo transfers. This may reflect clinicians’ tendency to transfer more embryos when the perceived prognosis is poorer, as well as a preference for Day 2 transfers when no surplus embryos are available for selection, in the hope that the *in vivo* environment may be more favorable for embryonic development. Although we adjusted for the number of transferred embryos in the multivariate model, and it remained an independent predictor, residual confounding is still possible. Future prospective studies with standardized transfer policies would help clarify this issue.

Although the proportion of fresh transfers was similar between MFP groups, the decision to cancel fresh transfer was not randomized. We have reported the reasons for cancellation and found no significant between−group differences, which somewhat mitigates selection bias. Nonetheless, residual confounding from unmeasured factors cannot be excluded.

Several limitations should be acknowledged. First, the retrospective design precludes causal inference. Second, although we performed a first-cycle sensitivity analysis to address within-patient correlation and time-related changes in ovarian reserve, residual confounding may persist. Third, the FET subgroup was underpowered; therefore, the null findings should not be interpreted as evidence of no association. Fourth, small subgroup sizes for Day 2 and blastocyst fresh transfers limit the reliability of these specific comparisons. Fifth, endometrial receptivity markers were not measured, so the proposed mechanism remains speculative.

## Conclusions

In summary, MFP ≥ 70% on the trigger day may be associated with higher fresh cycle LBR in low-prognosis patients under GnRH antagonist protocols. It provides a practical, ultrasound-based marker to optimize trigger timing for low-prognosis patients planning fresh transfers. Clinicians should prioritize achieving follicular synchrony within the 13-18mm window alongside traditional size thresholds.

## Data Availability

The raw data supporting the conclusions of this article will be made available by the authors, without undue reservation.

## Supplementary material

Supplementary Table 1 The distribution of patients according to the POSEIDON criteria based on the first oocyte-retrieval cycle stratified by the medium-sized follicles proportion (MFP).

Supplementary Table 2 Characteristics of embryos transferred and clinical outcomes of patients in the first oocyte-retrieval cycles stratified by the medium-sized follicles proportion (MFP). CPR, clinical pregnancy rate; EMR, early miscarriage rate; IR, implantation rate; LBR, live birth rate;

Supplementary Table 3 Multivariate logistic regression analysis of factors associated with live births in the first fresh embryo transfer cycles. MFP was entered as a continuous variable (per 1% increase) in the primary model. In a sensitivity analysis using the binary threshold (MFP ≥ 70% vs. < 70%), the association remained significant (adjusted OR = 2.88, 95% CI 1.74-4.89, P < 0.0001). All models were adjusted for the covariates listed. VIF values were < 2.5, indicating no significant multicollinearity. AFC, antral follicle count; CI, confidence intervals; MFP, medium-size follicle proportions; OR: odds ratio.
