# Supplementary material for: Medium-sized follicle proportion on the trigger day may be associated with higher live birth rate in fresh embryo transfer cycles among low-prognosis patients: a retrospective cohort study
Source: Front Endocrinol (Lausanne). 2026 Jul 15;17:1888870. doi: 10.3389/fendo.2026.1888870 (PMC13417633; doi:10.3389/fendo.2026.1888870)
Supplement: Supplementary Table 1 — The distribution of patients according to the POSEIDON criteria based on the first oocyte-retrieval cycle stratified by the medium-sized follicles proportion (MFP). [file Table1.pdf]

Supplementary Table 1. The distribution of patients according to the POSEIDON criteria based on the first oocyte-retrieval cycle stratified by the medium-sized follicles proportion (MFP).

	MFP < 70%	MFP ≥ 70%	P value
Group 1a	16 (5.02)	28 (6.28)	0.1940
Group 1b	33 (10.34)	50 (11.21)	/
Group 2a	25 (7.84)	37 (8.30)	/
Group 2b	44 (13.79)	53 (11.88)	/
Group 3	85 (26.65)	87 (19.51)	/
Group 4	116 (36.36)	191 (42.83)	/
