# Supplementary material for: Medium-sized follicle proportion on the trigger day may be associated with higher live birth rate in fresh embryo transfer cycles among low-prognosis patients: a retrospective cohort study
Source: Front Endocrinol (Lausanne). 2026 Jul 15;17:1888870. doi: 10.3389/fendo.2026.1888870 (PMC13417633; doi:10.3389/fendo.2026.1888870)
Supplement: Supplementary Table 2 — Characteristics of embryos transferred and clinical outcomes of patients in the first oocyte-retrieval cycles stratified by the medium-sized follicles proportion (MFP). CPR, clinical pregnancy rate; EMR, early miscarriage rate; IR, implantation rate; LBR, live birth rate; [file Table2.pdf]

Supplementary Table 2. Characteristics of embryos transferred and clinical outcomes of patients in the first oocyte-retrieval cycles stratified by the medium-sized follicles proportion (MFP).

| | MFP < 70% | MFP ≥ 70% | P value |
|---|---|---|---|
| Fresh transfer cycles (n) | 178 | 224 | / |
| Ratio of fresh transfers (%) | 55.80 (178/319) | 50.22(224/446) | 0.1279 |
| Endometrium thickness (mm) | 10.14 ± 2.22 | 9.85 ± 2.37 | 0.2158 |
| No. of embryos transferred (n) | **1.84 ± 0.63** | **1.65 ± 0.59** | **0.0025** |
| D2-embryo transfer | **2.39 ± 0.50** | **2.04 ± 0.45** | **0.0082** |
| D3-embryo transfer | 1.77 ± 0.58 | 1.64 ± 0.58 | 0.0518 |
| Blastocyst transfer | 1.27 ± 0.46 | 1.07 ± 0.27 | 0.3295 |
| Transferred embryo stage (%) | / | / | 0.0840 |
| D2-embryo transfer | 18.54(33/178) | 11.61(26/224) | / |
| D3-embryo transfer | 73.03(130/178) | 82.14(184/224) | / |
| Blastocyst transfer | 8.43(15/178) | 6.25(14/224) | / |
| Cycles with at least one good-quality embryo (%) | 95.51(170/178) | 97.77(219/224) | 0.2593 |
| D2-embryo transfer | 96.97(32/33) | 100(26/26) | > 0.9999 |
| D3-embryo transfer | 96.92(126/130) | 99.46(183/184) | 0.1640 |
| Blastocyst transfer | 80.00(12/15) | 71.43(10/14) | 0.6817 |
| CPR | **31.46 (56/178)** | **47.32 (106/224)** | **0.0013** |
| D2-embryo transfer | 27.27 (9/33) | 23.08 (6/26) | 0.7711 |
| D3-embryo transfer | **33.85 (44/130)** | **51.09 (94/184)** | **0.0024** |
| Blastocyst transfer | 20.00 (3/15) | 42.86 (6/14) | 0.2451 |
| IR | **21.04 (69/328)** | **32.16 (119/370)** | **0.0009** |
| D2-embryo transfer | 12.66 (10/79) | 11.32 (6/53) | 0.8175 |
| D3-embryo transfer | **24.35 (56/230)** | **35.43 (107/302)** | **0.0060** |
| Blastocyst transfer | 15.79 (3/19) | 40.00 (6/15) | 0.1392 |
| EMR | 28.57 (16/56) | 19.81 (21/106) | 0.2065 |
| D2-embryo transfer | 33.33 (3/9) | 16.67 (1/6) | 0.6044 |
| D3-embryo transfer | 29.55 (13/44) | 20.21 (19/94) | 0.2260 |
| Blastocyst transfer | 0.00 (0/3) | 16.67 (1/6) | > 0.9999 |
| LBR | **21.35 (38/178)** | **37.50 (84/224)** | **0.0005** |
| D2-embryo transfer | 18.18 (6/33) | 19.23 (5/26) | > 0.9999 |
| D3-embryo transfer | **22.31 (29/130)** | **33.04 (74/224)** | **0.0322** |
| Blastocyst transfer | 20.00 (3/15) | 35.71 (5/14) | 0.4270 |
| Ectopic pregnancy | 2 | 1 | / |

Footnote: CPR, clinical pregnancy rate; EMR, early miscarriage rate; IR, implantation rate; LBR, live birth rate.
