# Supplementary material for: Medium-sized follicle proportion on the trigger day may be associated with higher live birth rate in fresh embryo transfer cycles among low-prognosis patients: a retrospective cohort study
Source: Front Endocrinol (Lausanne). 2026 Jul 15;17:1888870. doi: 10.3389/fendo.2026.1888870 (PMC13417633; doi:10.3389/fendo.2026.1888870)
Supplement: Supplementary Table 3 — Multivariate logistic regression analysis of factors associated with live births in the first fresh embryo transfer cycles. MFP was entered as a continuous variable (per 1% increase) in the primary model. In a sensitivity analysis using the binary threshold (MFP ≥ 70% vs. < 70%), the association remained significant (adjusted OR = 2.88, 95% CI 1.74-4.89, P < 0.0001). All models were adjusted for the covariates listed. VIF values were < 2.5, indicating no significant multicollinearity. AFC, antral follicle count; CI, confidence intervals; MFP, medium-size follicle proportions; OR: odds ratio. [file Table3.pdf]

Supplementary Table 3. Multivariate logistic regression analysis of factors associated with live births in the first fresh embryo transfer cycles.

	OR (95% CI)	P value	Adjusted OR (95% CI)	P value
Female age (years)	**0.92(0.88-0.96)**	**0.0001**	**0.91(0.87-0.96)**	**0.0004**
AFC (n)	**1.13(1.05-1.22)**	**0.0009**	**1.09(1.01-1.19)**	**0.0311**
Endometrium thickness (mm)	**1.20(1.09-1.32)**	**0.0002**	**1.15(1.03-1.27)**	**0.0104**
The value of MFP (per 1% increase)	**1.01(1.00-1.02)**	**0.0365**	**1.02(1.01-1.03)**	**0.0013**
No. of retrieved oocytes (n)	**1.16(1.05-1.29)**	**0.0034**	1.07(0.94-1.22)	0.2860
No. of transferred embryos (n)	1.21(0.86-1.72)	0.2740	**1.78(1.10-2.92)**	**0.0195**
Transferred embryo stage	/	/	/	/
D3-embryo	Ref.	Ref.	Ref.	Ref.
D2-embryo	**0.47(0.22-0.91)**	**0.0333**	0.48(0.21-1.06)	0.0771
Blastocyst	0.78(0.32-1.76)	0.1832	1.06(0.37-2.76)	0.9105

Footnote: MFP was entered as a continuous variable (per 1% increase) in the primary model. In a sensitivity analysis using the binary threshold (MFP $\geq$ 70% vs. $<$ 70%), the association remained significant (adjusted OR = 2.88, 95% CI 1.74-4.89, $P < 0.0001$). All models were adjusted for the covariates listed. VIF values were $<$ 2.5, indicating no significant multicollinearity. Transferred embryo stage was categorized as D2, D3 (Ref., reference) and blastocyst. ORs were for each category relative to D3. AFC, antral follicle count; CI, confidence intervals; MFP, medium-size follicle proportions; OR: odds ratio.
